# Supplementary material for: Epigenetic aging and perceived psychological stress in old age
Source: Transl Psychiatry. 2022 Sep 26;12:410. doi: 10.1038/s41398-022-02181-9 (PMC9513097; doi:10.1038/s41398-022-02181-9)
Supplement: Supplementary file 1 — Supplemental Material [file 41398_2022_2181_MOESM1_ESM.docx]

**Epigenetic aging and perceived psychological stress in old age**

Valentin Max Vetter, MD^1, 2^, Johanna Drewelies, PhD^2^, Yasmine Sommerer, MSc^3^, Christian Humberto Kalies^1^, Vera Regitz-Zagrosek, MD^4, 5^, Lars Bertram, MD^3, 6^, Denis Gerstorf, PhD^2^, Ilja Demuth, PhD^1, 7^

^1^Charité – Universitätsmedizin Berlin, corporate member of Freie Universität Berlin and Humboldt-Universität zu Berlin, Department of Endocrinology and Metabolic Diseases (including Division of Lipid Metabolism), *Biology of Aging* working group, Augustenburger Platz 1, 13353 Berlin, Germany

^2^Department of Psychology, Humboldt University Berlin, Berlin, Germany

^3^Lübeck Interdisciplinary Platform for Genome Analytics (LIGA), University of Lübeck, Lübeck, Germany

^4^Institute for Gender in Medicine, Center for Cardiovascular Research, Charité - Universitätsmedizin Berlin, Corporate Member of Freie Universität Berlin, Humboldt - Universität zu Berlin and Berlin Institute of Health, Berlin, Germany

^5^Department of Cardiology, University Hospital Zürich, University of Zürich, Zürich, Switzerland

^6^Center for Lifespan Changes in Brain and Cognition (LCBC), Dept of Psychology, University of Oslo, Oslo, Norway

^7^Charité - Universitätsmedizin Berlin, BCRT - Berlin Institute of Health Center for Regenerative Therapies, Berlin, Germany

**Corresponding author:**

Ilja Demuth (Ph.D.)

Charité - Universitätsmedizin Berlin

Lipid Clinic at the Interdisciplinary Metabolism Center,

Biology of Aging Group

Augustenburger Platz 1

13353 Berlin

Email: [ilja.demuth@charite.de](mailto:ilja.demuth@charite.de)

Phone: ++49 30 450 569 143

FAX: ++49 30 450 566 904

Supplementary Material

**Supplementary Table 1: Sex-stratified descriptive statistics of the analyzed BASE-II participants of the GendAge study (n=1,100).**

|  | Women | | | | | |  | Men | | | | | |  |  |
| --- | --- | --- | --- | --- | --- | --- | --- | --- | --- | --- | --- | --- | --- | --- | --- |
|  | % | mean | SD | min | max | n |  | % | mean | SD | min | max | n |  | p-value |
| Chronological age (years) |  | 75.72 | 3.53 | 66.41 | 94.07 | 573 |  |  | 75.48 | 4.01 | 64.91 | 90.03 | 527 |  | 0.276 |
| Smoking (packyears) |  | 6.30 | 13.48 | 0.00 | 114.00 | 537 |  |  | 13.68 | 20.61 | 0.00 | 150.00 | 482 |  | <0.001 |
| BMI |  | 26.63 | 4.68 | 17.17 | 49.68 | 573 |  |  | 27.35 | 3.69 | 20.02 | 41.77 | 525 |  | 0.005 |
| Education (years) |  | 14.00 | 2.91 | 7.0 | 18.0 | 527 |  |  | 15.00 | 2.83 | 8.5 | 18.0 | 467 |  | <0.001 |
| Alcohol intake (yes) | 81.5 |  |  |  |  | 466 |  | 85.0 |  |  |  |  | 446 |  | 0.145 |
| Morbidity index |  | 1.36 | 1.51 | 0.00 | 9.00 | 492 |  |  | 1.43 | 1.58 | 0.00 | 9.00 | 462 |  | 0.478 |
| Frailty Score |  | 0.79 | 0.89 | 0.00 | 4.00 | 568 |  |  | 0.73 | 0.85 | 0.00 | 4.00 | 519 |  | 0.241 |
| CES-D |  | 13.82 | 3.91 | 0.00 | 31.00 | 568 |  |  | 13.25 | 3.46 | 2.00 | 35.00 | 521 |  | 0.010 |
| 7-CpG clock DNAmAA |  | -1.02 | 6.35 | -24.37 | 25.30 | 558 |  |  | 1.17 | 6.31 | -24.93 | 34.48 | 513 |  | <0.001 |
| Horvath's clock DNAmAA |  | -0.43 | 3.98 | -12.31 | 23.45 | 558 |  |  | 0.54 | 4.04 | -8.94 | 17.44 | 509 |  | <0.001 |
| Hannum's clock DNAmAA |  | -0.72 | 3.68 | -10.80 | 12.73 | 558 |  |  | 0.81 | 3.96 | -9.32 | 28.57 | 509 |  | <0.001 |
| PhenoAge DNAmAA |  | -0.48 | 5.39 | -16.54 | 25.80 | 558 |  |  | 0.62 | 5.39 | -13.51 | 20.94 | 509 |  | 0.001 |
| GrimAge DNAmAA |  | -1.30 | 2.93 | -10.82 | 10.71 | 558 |  |  | 1.47 | 3.27 | -8.17 | 12.85 | 509 |  | <0.001 |
| PSS |  | 2.11 | 0.68 | 1.00 | 4.50 | 516 |  |  | 2.04 | 0.59 | 1.00 | 4.38 | 490 |  | 0.082 |

Note: BMI: body mass index; CES-D Center for Epidemiologic Studies Depression Scale; PSS: Perceived Stress Scale; DNAmAA: DNA methylation age acceleration.

**Supplementary Table 2:** **Sex-stratified multiple linear regression analyses of morbidity index, frailty score and CES-D on Cohen’s PSS in older BASE-II participants of the GendAge study.** Linear regression models were adjusted for covariates. Model 1: no adjustment; Model 2: chronological age; Model 3: Model 2 + smoking (packyears), alcohol (yes/no), BMI, and education.

|  |  | Women | | | | |  | Men | | | | |
| --- | --- | --- | --- | --- | --- | --- | --- | --- | --- | --- | --- | --- |
|  | Model | Estimate | SE | p-value |  | n |  | Estimate | SE | p.value |  | n |
| Morbidity Index | 1 | 0.268 | 0.068 | <0.001 | *** | 444 |  | 0.163 | 0.082 | 0.048 | * | 430 |
|  | 2 | 0.264 | 0.068 | <0.001 | *** | 444 |  | 0.166 | 0.082 | 0.044 | * | 430 |
|  | 5 | 0.310 | 0.072 | <0.001 | *** | 397 |  | 0.177 | 0.088 | 0.045 | * | 355 |
| Fried's Frailty Phenotype | 1 | 0.191 | 0.036 | <0.001 | *** | 511 |  | 0.167 | 0.042 | <0.001 | *** | 484 |
|  | 2 | 0.186 | 0.036 | <0.001 | *** | 511 |  | 0.166 | 0.041 | <0.001 | *** | 484 |
|  | 5 | 0.204 | 0.037 | <0.001 | *** | 451 |  | 0.154 | 0.045 | 0.001 | *** | 401 |
| CES-D | 1 | 1.109 | 0.155 | <0.001 | *** | 512 |  | 0.549 | 0.160 | 0.001 | *** | 485 |
|  | 2 | 1.110 | 0.156 | <0.001 | *** | 512 |  | 0.548 | 0.161 | 0.001 | *** | 485 |
|  | 5 | 1.005 | 0.161 | <0.001 | *** | 453 |  | 0.567 | 0.177 | 0.001 | *** | 401 |

Note: SE: standard error, CES-D: Center for Epidemiologic Studies Depression Scale.

**Supplementary Table 3: Sex-stratified multiple linear regression of Cohen’s PSS on DNAmAA of five epigentic clocks and covariates.** Model 1: no covariates; Model 2: smoking (packyears), alcohol intake (yes/no), BMI, education, and genetic ancestry.

|  |  | Women | | | | |  | Men | | | | |
| --- | --- | --- | --- | --- | --- | --- | --- | --- | --- | --- | --- | --- |
|  | Model | Estimate | SE | p-value |  | n |  | Estimate | SE | p-value |  | n |
| 7-CpG DNAmAA | 1 | -0.302 | 0.264 | 0.253 |  | 502 |  | -0.196 | 0.315 | 0.534 |  | 478 |
|  | 2 | -0.141 | 0.293 | 0.631 |  | 415 |  | -0.153 | 0.367 | 0.678 |  | 358 |
| Horvath's DNAmAA | 1 | -0.273 | 0.167 | 0.102 |  | 502 |  | -0.263 | 0.201 | 0.191 |  | 474 |
|  | 2 | -0.138 | 0.183 | 0.450 |  | 415 |  | -0.209 | 0.242 | 0.389 |  | 356 |
| Hannum's DNAmAA | 1 | -0.165 | 0.154 | 0.286 |  | 502 |  | 0.088 | 0.196 | 0.654 |  | 474 |
|  | 2 | -0.042 | 0.175 | 0.811 |  | 415 |  | 0.144 | 0.233 | 0.536 |  | 356 |
| PhenoAge DNAmAA | 1 | -0.148 | 0.229 | 0.518 |  | 502 |  | -0.668 | 0.265 | 0.012 | * | 474 |
|  | 2 | 0.165 | 0.240 | 0.493 |  | 415 |  | -0.513 | 0.300 | 0.088 |  | 356 |
| GrimAge DNAmAA | 1 | -0.007 | 0.123 | 0.953 |  | 502 |  | 0.040 | 0.162 | 0.805 |  | 474 |
|  | 2 | 0.066 | 0.131 | 0.611 |  | 415 |  | -0.039 | 0.177 | 0.826 |  | 356 |

Note: DNAmAA: DNA methylation age acceleration; SE: Standard Error.

**Supplementary Table 4: Overview over literature of the field.**

| Study | Sample size,  Study | Mean age (SD; range) | Female sex (%) | Type of stress or trauma (instrument) | Epigenetic Parameter | Main findings |
| --- | --- | --- | --- | --- | --- | --- |
| Simons et al., 2016^1^ | N=100  FACHS | 48.5 (9.2) | 100 | SES (income and financial pressure) Childhood trauma, Lifestyle (tobacco, alcohol intake, exercise, diet, BMI) | Hannum DNAmAA (residuals) | Association with lower income, higher financial pressure.  No association with childhood trauma and lifestyle. |
| Fiorito et al., 2017^2^ | N=5111  EPIC Italy, MCCS, TILDA | 57.3 | 48.0 | SES (educational attainment, occupational position, income) | Horvath,  Hannum (cell count adjusted residuals) | Metanalysis: Association between higher Horvath and Hannum DNAmAA and lower SES. |
| Hughes et al., 2018 ^3^ | N=1099  UK Household Longitudinal Study | 58.4 (14.9; 28-98) | 57.6 | SES (current income and employment, education, income and unemployment across a 12-year period, and childhood social class) | Horvath  Hannum | Association between low SES during childhood and higher DNAmAA. No associations between current SES and DNAmAA. |
| Lawn et al., 2018^4^ | N=989 (twice) + N=773  ALSPAC, NSHD | 28.65 (5.54)  47.44 (4.42)  53.44 (0.26) | 100 | SES (father’s occupational social class and highest current occupational social class as “high”/”low”), Psychosocial adversity during childhood | Horvath | Association between sexual abuse during childhood and higher DNAmAA. No association between SES and DNAmAA. |
| McCrory et al., 2019 ^5^ | N=490  TILDA | 62.2 (8.3; 50-87) | 50.2 | SES (social class, education and income tertiles) | Horvath  Hannum  PhenoAge | No association between DNAmAA and SES. |

References:

1. Simons RL, Lei MK, Beach SR, Philibert RA, Cutrona CE, Gibbons FX *et al.* Economic hardship and biological weathering: the epigenetics of aging in a US sample of black women. *Social Science & Medicine* 2016; **150:** 192-200.

2. Fiorito G, Polidoro S, Dugué P-A, Kivimaki M, Ponzi E, Matullo G *et al.* Social adversity and epigenetic aging: a multi-cohort study on socioeconomic differences in peripheral blood DNA methylation. *Scientific reports* 2017; **7**(1)**:** 1-12.

3. Hughes A, Smart M, Gorrie-Stone T, Hannon E, Mill J, Bao Y *et al.* Socioeconomic position and DNA methylation age acceleration across the life course. *American journal of epidemiology* 2018; **187**(11)**:** 2346-2354.

4. Lawn RB, Anderson EL, Suderman M, Simpkin AJ, Gaunt TR, Teschendorff AE *et al.* Psychosocial adversity and socioeconomic position during childhood and epigenetic age: analysis of two prospective cohort studies. *Human molecular genetics* 2018; **27**(7)**:** 1301-1308.

5. McCrory C, Fiorito G, Cheallaigh CN, Polidoro S, Karisola P, Alenius H *et al.* How does socio-economic position (SEP) get biologically embedded? A comparison of allostatic load and the epigenetic clock (s). *Psychoneuroendocrinology* 2019; **104:** 64-73.
